# Symmetry in the Enlargement Rates of Large Hypertransmission Defects between Eyes in Age-Related Macular Degeneration

**DOI:** 10.1016/j.xops.2026.101333

**Published:** 2026-07-22

**Authors:** Sara Beqiri, Gissel Herrera, Mengxi Shen, Alessandro Berni, Omar S. El-Mulki, Omar Badla, Winston Lam, Eduardo Herrera, Niccolò Ascioti, Jillene Moxam, Scott Shuldiner, Nithya Boopathiraj, Yuxuan Cheng, Viet Hoan Le, Omer Trivizki, Robert C. O'Brien, Ruikang K. Wang, Giovanni Gregori, Philip J. Rosenfeld

**Affiliations:** 1 Department of Ophthalmology, Bascom Palmer Eye Institute, University of Miami Miller School of Medicine, Miami, Florida; 2 Department of Neurosciences, Psychology, Drug Research, and Child Health, Eye Clinic, University of Florence, AOU Careggi, Florence, Italy; 3 College of Medicine, University of Florida, Gainesville, Florida; 4 Department of Bioengineering, University of Washington, Seattle, Washington; 5 Department of Ophthalmology, Tel Aviv Medical Center, Tel Aviv, Israel; 6 Department of Ophthalmology, University of Washington, Seattle, Washington

**Keywords:** Swept-source OCT angiography (SS-OCTA), Age-related macular degeneration (AMD), Geographic atrophy (GA), Symmetry, Clinical trial design

## Abstract

**Purpose:**

The symmetry of macular atrophy growth rates between eyes of patients with bilateral nonexudative age-related macular degeneration (AMD) was assessed to determine if both eyes could be used in clinical trials to test therapies that might slow the growth of atrophy.

**Design:**

Retrospective review of prospectively collected swept-source OCT angiography images.

**Subjects:**

Patients with bilateral nonexudative AMD with ≥1 large hypertransmission defect (hyperTD) in both eyes were included.

**Methods:**

All large hyperTDs, defined by a greatest linear dimension ≥250 μm, were identified by 2 independent graders and annotated using a semiautomated algorithm. Growth rates were assessed using the area, square root (sqrt) area, and the perimeter-adjusted growth-rate strategies. Baseline lesion sizes were stratified using a size breakpoint of 0.9 disc area (DA). Intereye comparisons of annual hyperTD growth rates were performed. Power analyses were calculated to determine the sample sizes needed for different clinical trial designs.

**Main Outcome Measures:**

Intereye concordance of growth rates was evaluated using Lin concordance correlation coefficients, and inter eye growth-rate agreement was evaluated using Bland–Altman analyses. Sample sizes for a 2-arm clinical trial design were calculated to detect a 30% reduction in growth rates in the treatment arm with 80% power and a 2-sided α = 0.05.

**Results:**

Sixty-four subjects (128 eyes) were followed for a mean of 4.2 ± 1.9 years, providing 942 annualized growth-rate intervals. Prior to stratification by baseline size of 0.9 DA, intereye concordance was high (r_c_ = 0.76) for the total area growth rate, but lower for the sqrt and perimeter-adjusted growth rates (r_c_ = 0.51, r_c_ = 0.60, respectively). Upon stratification, lesions <0.9 DA showed low correlation (0.39–0.46) across the 3 growth rates, but lesions ≥0.9 DA showed high correlation regardless of the transformation strategy (0.73–0.78).

**Conclusions:**

High intereye concordance in hyperTD growth rates was observed for lesions ≥0.9 DA using 3 different growth-rate strategies. Regardless of the growth-rate strategy used, recruitment of fellow eyes in a paired-eye study for localized treatments or both eyes for systemic treatments resulted in a marked reduction in sample sizes, with improved clinical trial efficiency.

**Financial Disclosure(s):**

Proprietary or commercial disclosure may be found in the Footnotes and Disclosures at the end of this article.

Geographic atrophy (GA) is the late stage of nonexudative age-related macular degeneration (AMD) associated with the loss of photoreceptors, retinal pigment epithelium (RPE), and choriocapillaris (CC), and GA is a major cause of irreversible vision loss among the elderly worldwide.^1^^,^^2^ Numerous studies have investigated structural and angiographic features in the macula associated with faster enlargement rates (ERs) of GA.^3^, ^4^, ^5^, ^6^, ^7^, ^8^, ^9^ These macular features include multifocality, noncircularity, nonfoveal location, the attenuation or loss of the surrounding photoreceptors, the perilesional accumulation of basal laminar deposits, the presence of reticular pseudodrusen, also known as subretinal drusenoid deposits, and an increase in macular CC flow deficits, which are presumed to correspond to decreased CC perfusion.

While AMD is a complex genetic disease influenced by environmental factors, one possible strategy to distinguish between the role of genetic and nongenetic influences on the ER of GA would be to study the symmetry of ERs between eyes of the same patient. An important reason to study the symmetry of ERs is to determine if these fellow eyes could be used as controls in clinical trials in lieu of a separate control group. If fellow eyes could be used as controls, then fewer subjects would be needed in a clinical trial, resulting in significant cost savings while having control eyes that are age-matched and genetically matched with the treatment eyes.

Previously, several studies have investigated the symmetry of GA ERs between fellow eyes of the same patient and reported correlation coefficients as high as 0.86.^5^ While a strong linear correlation is necessary, it is not sufficient by itself to establish symmetry. In the present study, we define intereye symmetry as the presence of a strong linear relationship, comparable mean growth rates (absence of systematic bias), and low variability between the eyes, aspects that have not previously been addressed in detail.^5^^,^^10^ Some of the variability from previous studies might be in part explained by the use of area measurements, because it is well known that ERs are highly dependent on the baseline characteristics such as lesion area, lesion circularity, and lesion multifocality. This could be addressed by using a square root (sqrt) transformation of the area measurements because we previously showed that this sqrt transformation strategy greatly diminished the dependence of ER on baseline lesion area measurements.^11^ Furthermore, Shen et al^6^ claimed to improve upon the sqrt transformation strategy by devising the perimeter-adjusted transformation strategy that appeared to further decrease the impact of baseline lesion area, multifocality, and circularity on the ERs of GA lesions. Herrera et al^12^ then evaluated the suitability of each growth measurement strategy according to baseline GA area, and found the growth rate of eyes with baseline GA area <0.9 disc area (DA) was best measured using either the perimeter-adjusted strategy or the sqrt area strategy, whereas eyes with baseline area ≥0.9 DA can be followed using any of the total area, sqrt area, or perimeter-adjusted growth strategies. To date, these transformation strategies have not been used to study the symmetry of GA ERs.

Moreover, these previous studies investigating the symmetry of ERs used color fundus imaging (CFI) or fundus autofluorescence (FAF) imaging to assess GA. In this current study, we use OCT imaging, which is considered the gold standard for imaging the anatomy of GA.^13^^,^^14^

A comparison of GA area measurements by FAF and en face OCT revealed good correlation between the modalities; however, OCT consistently measured lesions as smaller across all sizes (mean area 6.82 mm^2^).^15^ In contrast, Yehoshua et al^16^ found that OCT yielded larger baseline GA areas than fluorescein angiography or FAF-based measurements (mean area 2.43 mm^2^) but resulted in lower subsequent growth rates. When comparing FAF with color fundus photographs, Domalpally et al^17^ revealed low agreement at baseline (43%), which improved to (81%) by year 5, demonstrating that color fundus photography is even less suitable for detection of early lesions. These differences highlight the greater sensitivity of OCT to detect early and small areas of atrophy and the ability of OCT to more precisely delineate borders, which reduces overestimation of ERs compared with FAF or fluorescein angiography.

To measure GA using OCT, we used the appearance and growth of large choroidal hypertransmission defects (hyperTDs),^18^ and these large hyperTDs were shown to correlate with GA detected using both CFI and FAF images.^15^^,^^16^ Images of these large hyperTDs were obtained by using an *en face* projection of a slab positioned beneath the RPE.^18^ On these structural *en face* images, regions with RPE attenuation or loss appear bright, allowing us to detect the onset and progression of early lesions that progressed to typical GA.^19^, ^20^, ^21^, ^22^

The onset and progression of these hyperTDs were studied using dense swept-source OCT angiography (SS-OCTA) scanning patterns in eyes with intermediate AMD and the earliest detectable change that persisted and progressed to GA had a greatest linear dimension of ≥250 μm on *en face* OCT imaging.^20^^,^^23^ This finding was then independently reproduced by Nanegrungsunk et al,^24^ and these persistent hyperTDs are now referred to as large hyperTDs. The advantage of using *en face* OCT to study disease progression in eyes with intermediate AMD is that the same OCTA scan can be used to monitor the other structural features, as well as CC perfusion, that have been associated with more rapid GA ERs. In this study, we compare the annual ERs of these hyperTDs in subjects with bilateral large hyperTDs monitored prospectively with SS-OCTA scans using different transformation strategies to measure their growth rates and assess the concordance between eyes so that both eyes of the same subjects could be used in clinical trials.

## Methods

Patients with AMD were enrolled in an ongoing prospective, observational, swept-source OCT (SS-OCT) imaging study at the Bascom Palmer Eye Institute. The study was approved by the Institutional Review Board of the University of Miami Miller School of Medicine, and all patients signed an informed consent for this prospective SS-OCT study. The study was performed in accordance with the tenets of the Declaration of Helsinki and complied with the Health Insurance Portability and Accountability Act of 1996.

### Participants

A retrospective review of subjects prospectively enrolled in the OCT imaging study was performed to identify eyes with treatment-naive, nonexudative AMD and ≥1 large choroidal hyperTD measuring ≥250 μm in greatest linear dimension. For this study of bilateral symmetry of hyperTD growth rates, patients were required to have large hyperTDs in both eyes with at least 12 ± 1 months of follow-up from their baseline visit to their last available visit. All included eyes had an axial length <26.5 mm or refractive power of < -6 D.

The exclusion criteria at baseline included a history of exudative macular neovascularization, diabetic retinopathy, vascular occlusions, central serous chorioretinopathy, retinal diseases associated with drusen-like deposits, such as Stargardt disease or vitelliform dystrophy, pathological myopia, and any vitreoretinal interface diseases distorting the macular anatomy or previous vitreoretinal surgery. Development of exudation due to macular neovascularization, retinal pigment epithelial tears, and initiation of intravitreal VEGF inhibitor or pegcetacoplan injections were considered criteria for exclusion of further visits.

### Imaging Protocol

All patients underwent SS-OCTA imaging (PLEX Elite 9000, Carl Zeiss, Meditec Inc) acquired by 1 of 2 trained imaging technicians. The swept-source laser has a central wavelength of 1050 nm and bandwidth of 100 nm, providing an axial resolution of ∼5 μm in tissue and an estimated transverse resolution of ∼15 μm at the retinal surface. All scans were acquired at a scanning rate of 100 kHz.

Fovea-centered scans were acquired for all eyes using the 6 × 6-mm and 12 × 12-mm angiographic scan patterns. All scans consisted of 500 A-scans per B-scan and 500 B-scans, with each B-scan repeated twice at every position, resulting in uniform spacing between A-scans of 12 μm in the 6 × 6-mm pattern, and 24 μm in the 12 × 12-mm pattern. Scans with a signal strength <7 as indicated on the instrument, as well as those with significant motion artifacts as reviewed by the 2 graders were excluded, and where several images were available for the same-visit, the highest quality were used.

### Grading of hyperTDs

**Figure 1:**
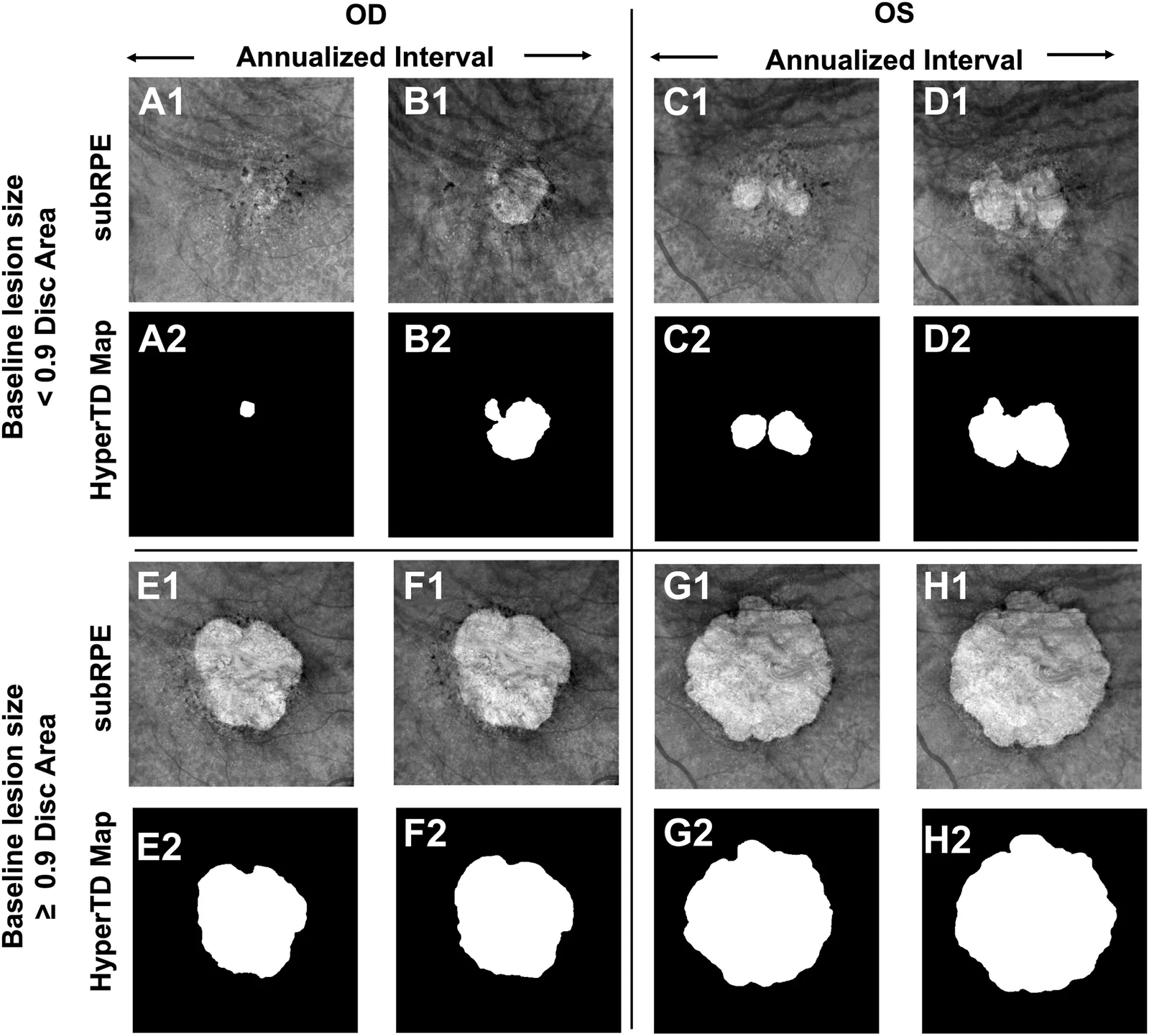
Two examples of annualized bilateral growth-rate intervals of large hyperTDs from a longitudinal natural history studying the symmetry of hyperTD growth in patients with AMD. **A1-D1**, Right eye and OS from a patient with bilateral large hyperTDs that measured <0.9 DA. **A1-D1** and **E1-H1,** Structural *en face* subRPE slabs. **A2-D2** and **E2-H2,** Binarized maps derived from manual annotation of large hyperTDs on the corresponding *en face* subRPE slab. **A1-D1**, Bilateral hyperTD progression over an annualized interval in eyes with baseline lesion area <0.9 DAs (2.29 mm^2^), measuring 0.14 mm^2^ OD and 1.67 mm^2^ OS. Hypertransmission defect growth rates measured using the total area, sqrt area, and perimeter-adjusted growth-rate strategies were 2.08 mm^2^/year, 1.11 mm/year, and 0.46 mm/year in OD and 2.30 mm^2^/year, 0.70 mm/year, and 0.27 mm/year in OS, demonstrating intereye asymmetry particularly for the sqrt and perimeter-adjusted strategies, defined as any difference outside the lower confidence limit of the lower LoA or the upper confidence limit of the upper LoA (Figs 3 and 5). **E1-H1**, Bilateral hyperTD progression over an annualized interval, in which atrophy in both eyes is more advanced and baseline lesion area is ≥0.9 DA, measuring 6.76 mm^2^ in OD and 11.27 mm^2^ in OS. Hypertransmission defect growth rates measured using the total area, sqrt area, and perimeter-adjusted growth-rate strategies were 1.18 mm^2^/year, 0.22 mm/year, and 0.11 mm/year in OD and 2.03 mm^2^/year, 0.29 mm/year, and 0.14 mm/year in OS, demonstrating improved intereye alignment when using the sqrt area and perimeter-adjusted strategies. AMD = age-related macular degeneration; DA = disc area; hyperTD = hypertransmission defect; LoA = limits of agreement; OD = right eye; OS = left eye; sqrt = square root; subRPE = subretinal pigment epithelium.

Large hyperTDs were defined as regions of increased brightness with a greatest linear dimension measuring ≥250 μm on the *en face* structural sub-RPE slabs obtained using segmentation boundaries at 64 to 400 μm beneath the Bruch's membrane (Fig 1). Corresponding B-scans were used to confirm the presence of choroidal hyperTDs due to the attenuation or loss of the RPE and outer retina.^19^^,^^20^^,^^22^, ^23^, ^24^

Grading was performed by 2 independent graders (S.B. and G.H.), with any disagreements adjudicated by a third senior grader (P.J.R). Lesions were manually annotated using a semiautomated algorithm developed by Chu et al.^25^ All available visits for each eye were reviewed and annotated using this software. Every lesion was assigned a unique identifier that was maintained throughout the follow-up to ensure consistent tracking of the same hyperTDs across visits. When lesions merged, their identifiers were combined.

Reviews and annotations were performed using the 6 × 6-mm scan pattern. If a hyperTD exceeded this field of view at any point during the follow-up, a 12 × 12-mm pattern was used from that visit onward. Eyes in which lesions exceeded the 12 × 12-mm field of view were censored from follow-up.

### Statistical Methods

All visit intervals of 1 year ± 1 month with available bilateral data were included. Herrera et al^12^ investigated hyperTD growth rates in 221 eyes with dry AMD and lesion sizes ranging from 0.04 to 51.62 mm^2^, demonstrating that smaller and larger lesions exhibit distinct growth patterns driven by differences in baseline area, perimeter, focality, and circularity. Based on these findings, they recommended the use of different growth-rate measurement strategies to account for this complexity. Accordingly, we applied their identified breakpoint of 0.9 DA to stratify lesions by baseline hyperTD area for each interval and included only intervals in which both eyes belonged to the same baseline size category. We then assessed the symmetry of annualized hyperTD growth rates using 3 growth-rate strategies: total area growth (mm^2^/year;) sqrt total area growth (mm/year), and perimeter-adjusted growth (mm/year).

We assessed agreement and symmetry between right eye (OD) and left eye (OS) hyperTD growth-rate outcomes using Bland–Altman analysis^26^ and Lin concordance correlation^27^ coefficient (r_c_). In contrast to Pearson correlation coefficient, which evaluates only the strength of the linear relationship, Lin concordance correlation coefficient accounts for the distance of the ordinary least squares regression line from the line of equality through the origin. This addresses the shortcomings of standard correlation coefficients with respect to agreement analysis where one measurement can be systematically much higher than the other and yield a high correlation, thus providing a more appropriate assessment of true symmetry. Complementary Bland–Altman plots were used to visually display the absence of systematic bias and the spread of the differences in growth rates. Due to its appropriateness and convenience as a single index, we used Lin concordance correlation coefficient as a direct quantitative indicator of intereye symmetry.

Cluster bootstrap resampling with patients as the resampling unit was used to account for clustering of fellow eyes as well as repeated measurements from the same eye. We also calculated the sample sizes for a 2-arm, 1-year randomized clinical trial (RCT) for reducing growth of large hyperTDs for each growth-rate outcome using observed means and standard deviations assuming a normal distribution for the difference between the treatment arm and the untreated arm, a paired *t* test, 80% power to detect a 30% reduction in ERs in the treatment arm compared with the untreated arm, 2-sided α = 0.05, and 1:1 treatment allocation with fellow eyes randomized to different arms. We then compared these sample sizes to the sample sizes for the unpaired design (i.e., with only 1 eye enrolled per participant) in Herrera et al.^12^ Furthermore, to address trial designs where treatment is assigned at the patient level instead of the eye level (i.e., fellow eyes receive the same treatment), we calculated the sample sizes for a 2-arm, 1-year RCT for each growth outcome assuming a 2-sample *t* test using observed means and standard deviations and multiplied the numbers by a factor of (1 + r_c_), where r_c_ represents the intereye correlation between OD and OS.^28^^,^^29^ *P* values were obtained via confidence interval (CI) inversion and a 2-sided *P* value <0.05 was considered statistically significant. All statistical analyses were performed using R version 4.5.2 (R Foundation for Statistical Computing) with the boot, boot.pval, pwr, segmented, SimplyAgree, and tidyverse packages.^30^, ^31^, ^32^, ^33^, ^34^, ^35^, ^36^, ^37^

## Results

A total of 146 eyes from 73 patients with bilateral nonexudative AMD and large hyperTDs were identified for this study. Nine patients were excluded due to not having ≥1 matched interval 1 year ± 1 month in both eyes.

The final cohort consisted of 128 eyes from 64 patients, and 46 (71.9%) were female. The mean age at baseline was 75.4 ± 8.3 years, and they were followed for a mean of 4.2 ± 1.9 years. When a breakpoint of 0.9 DA, as defined by Herrera et al,^12^ was used to stratify lesions according to baseline hyperTD area in both eyes, 5 additional patients were excluded because their fellow eyes belonged to different size categories across all available follow-up intervals.

### Baseline hyperTD Features

Extraction of annualized follow-up intervals yielded a total of 942 paired intervals from 128 eyes of 64 subjects. After stratifying on baseline total hyperTD area, there were 348 paired intervals from 33 participants with bilateral total hyperTD area <0.9 DA, and 438 paired intervals from 34 participants with bilateral total hyperTD area ≥0.9 DA. Eight participants contributed intervals to both subsets at different points during the follow-up periods.

At baseline, across all eyes (OD and OS) and annual intervals, the mean total hyperTD area was 4.17 ± 4.38 (range: 0.04 – 30.30) mm^2^, mean perimeter was 13.41 ± 9.42 (range: 0.73 – 52.26) mm, mean circularity was 0.35 ± 0.25 (0.04 – 0.95), and the mean number of lesions was 4.88 ± 4.16 (1 – 25). Baseline characteristics by OD and OS are summarized by Table 1.

**Table 1:** Baseline hyperTD Lesion Characteristics by OD and OS, Presented as Mean (SD) [Min; Max]

	OD	OS	*P* Value
	N = 471∗	N = 471†	
Initial total area (mm^2^)	4.26 (4.39) [0.04;30.30]	4.08 (4.37) [0.05;22.39]	0.57
Initial total perimeter (mm)	13.85 (9.72) [0.73;52.26]	12.96 (9.09) [0.87;47.50]	0.29
Initial circularity index	0.33 (0.24) [0.04;0.94]	0.36 (0.25) [0.04;0.95]	0.29
Initial number of lesions	5.06 (4.15) [1.00;22.00]	4.70 (4.16) [1.00;25.00]	0.33
Total area growth (mm^2^/yr)	1.13 (0.97) [<0.01;5.96]	1.05 (0.86) [<0.01;4.96]	0.24
Sqrt area growth (mm/yr)	0.28 (0.20) [<0.01;1.56]	0.26 (0.16) [<0.01;0.94]	0.39
Perim-Adj growth (mm/yr)	0.08 (0.06) [<0.01;0.47]	0.08 (0.05) [<0.01;0.29]	0.94

hyperTD = hypertransmission defect; Min = minimum; Max = maximum; OD = right eye; OS = left eye; Perim-Adj = perimeter-adjusted; SD = standard deviation; Sqrt = square root.

∗ The number of annual intervals from OD of 64 subjects.

† The number of annual intervals from OS of 64 subjects.

### Intereye Concordance Correlation Coefficients

**Figure 2:**
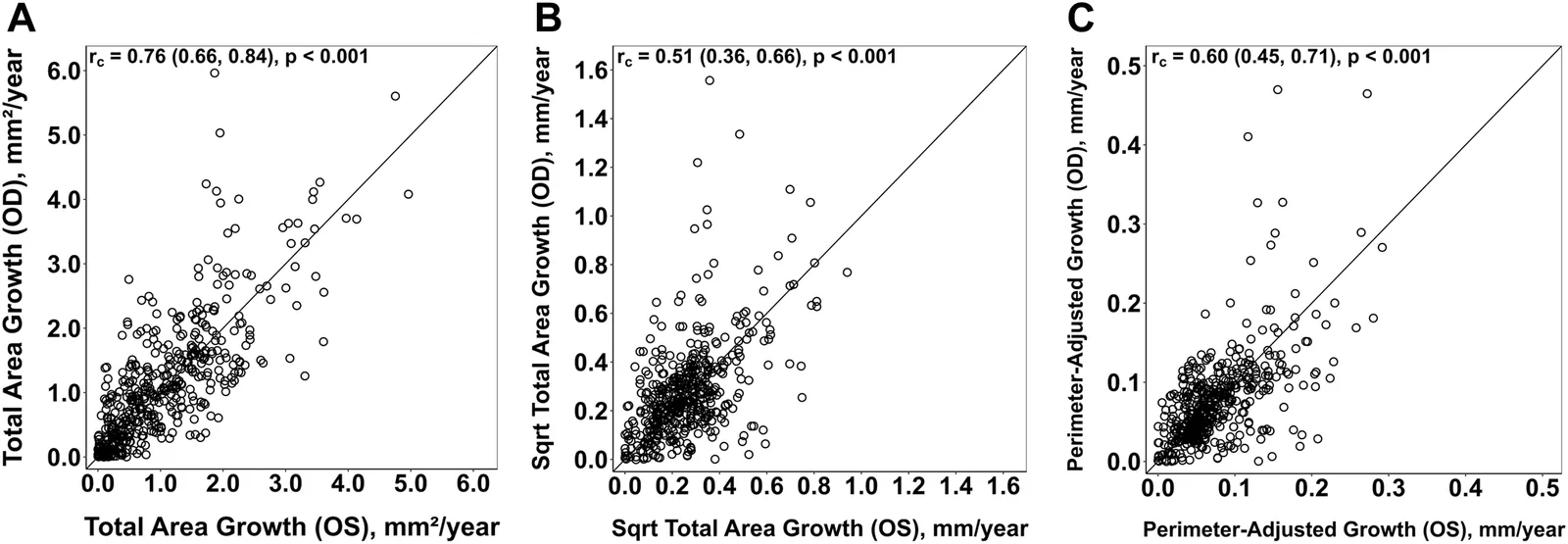
Scatterplots showing intereye concordance of annualized growth rates of large hyperTDs across all baseline lesion sizes using 3 growth-rate strategies. The y-axis shows growth rates for the OD, and the x-axis shows those for the OS. The diagonal line denotes the line of equality (slope = 1), indicating perfect agreement between OD and OS and a concordance correlation coefficient (r_c_) of 1. Each point represents an annualized growth-rate interval of 1 year ± 1 month. Confidence intervals and *P* values for Lin concordance correlation coefficients were obtained using cluster bootstrap resampling. **A,** Right eye–OS concordance for the total area growth strategy (mm^2^/year), with a concordance correlation coefficient of r_c_ = 0.76 (*P* < 0.001), indicating strong, statistically significant concordance. **B,** Right eye–OS concordance for the square root (sqrt) total area strategy (mm/year), with r_c_ = 0.51 (*P* < 0.001); **C,** OD–OS concordance for the perimeter-adjusted strategy (mm/year), with r_c_ = 0.60 (*P* < 0.001); both indicate statistically significant but moderate concordance. hyperTD = hypertransmission defect; OD = right eye; OS = left eye.

In an initial analysis, all annualized intervals were plotted irrespective of baseline lesion size using 3 growth strategies. Figure 2 shows the intereye concordance scatterplots, demonstrating a strong correlation for total area growth (r_c_ = 0.76 [95% CI: 0.66 to 0.84]; *P* < 0.001). In comparison, sqrt area growth and perimeter-adjusted area growth showed only moderate correlations with correlation coefficients of r_c_ = 0.51 (95% CI: 0.36 to 0.66) and r_c_ = 0.60 (95% CI: 0.45 to 0.71), respectively (*P* < 0.001 for both).

As Herrera et al^12^ reported, different growth-rate transformations are not interchangeably suitable for lesions of all sizes. To understand whether this observed difference in concordance was attributable to the growth-rate strategy used, further analyses were performed applying the 3 strategies in lesions categorized by the previously defined 0.9 DA breakpoint (Fig 4). For smaller lesions (<0.9 DA), intereye concordance was weak to moderate across all 3 growth strategies (total area r_c_ = 0.46 [95% CI: 0.27 to 0.66], sqrt area r_c_ = 0.39 [95% CI: 0.14 to 0.58], perimeter-adjusted r_c_ = 0.41 [95% CI: 0.08 to 0.62]) with a noticeable reduction in correlation for the total area growth strategy compared with the unstratified analysis (Fig 4A–C). In contrast, for larger lesions (≥0.9 DA), concordance was consistently strong across all strategies (total area r_c_ = 0.73 [95% CI: 0.57 to 0.84], sqrt area r_c_ = 0.73 [95% CI: 0.54 to 0.86], perimeter-adjusted r_c_ = 0.78 [95% CI: 0.65 to 0.89]), with improved performance of the sqrt total area growth and perimeter-adjusted growth strategies (Fig 4D–F).

In a secondary analysis, we sought to determine whether the observed high concordance between fellow eyes could be explained by similarity in the genetics and environmental exposures in the same patient. We performed random pairing between OD and OS unrelated eyes from different patients and using the same stratification by baseline lesion size as the primary analysis, we found near-zero concordance in growth rates. For intervals of all baseline sizes combined, the concordance coefficients were 0.01 (–0.07, 0.06), *P* = 0.83 for total area growth, 0.03 (–0.07, 0.06), *P* = 0.31 for sqrt growth, and –0.01 (–0.07, 0.06), *P* = 1.00 for perimeter-adjusted growth. For intervals <0.9 DA at baseline, concordance coefficients were –0.02 (–0.09, 0.09), *P* = 1.00; –0.03 (–0.09, 0.09), *P* = 1.00; –0.04 (–0.09, 0.09), *P* = 1.00, whereas for intervals ≥0.9 DA at baseline these were 0.01 (–0.10, 0.10), *P* = 0.77; 0.06 (–0.10, 0.09), *P* = 0.21; 0.03 (–0.11, 0.09), *P* = 0.58 for the total area, sqrt area and perimeter-adjusted area growth rates respectively.

### Intereye Agreement Analyses

**Figure 3:**
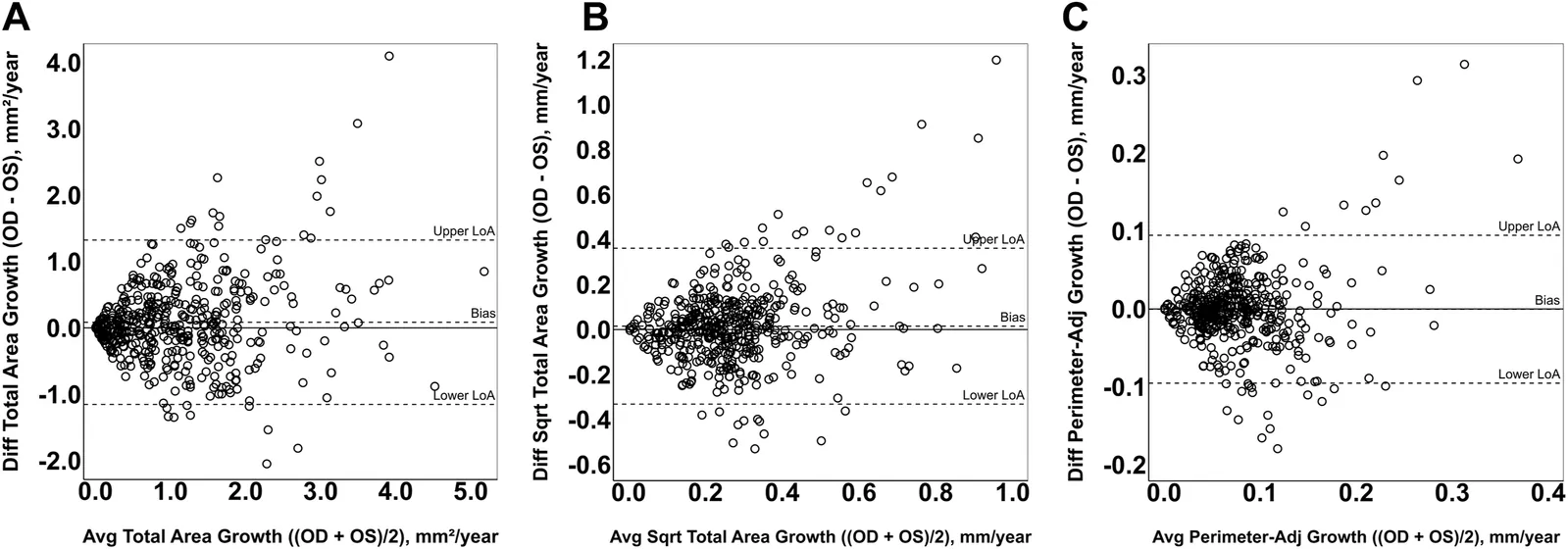
Bland–Altman plots showing intereye agreement in annualized growth rates of large hyperTDs across all baseline lesion sizes using 3 growth-rate strategies. The y-axis shows the difference in growth rate between the OD and OS, and the x-axis shows the mean growth rate of both eyes. Each dot represents an annualized growth-rate interval of 1 year ± 1 month. The central dashed line (“Bias”) denotes the mean intereye difference. The upper and lower dotted lines (“Upper LoA” and “Lower LoA”) represent the 95% limits of agreement, calculated as Bias ± 1.96 × SD_differences_. **A,** Intereye agreement using the total area growth strategy; **B,** intereye agreement using the sqrt total area strategy; and **C,** intereye agreement using the perimeter-adjusted strategy. Because the growth rates are expressed in different units, direct comparison of OD–OS agreement across strategies is not appropriate. hyperTD = hypertransmission defect; LoA = limits of agreement; OD = right eye; OS = left eye; SD = standard deviation; sqrt = square root.

Bland–Altman analysis was used to assess intereye agreement across all annualized intervals, first including all baseline lesion sizes and subsequently stratified using the 0.9 DA breakpoint (Figs 3 and 5). When applying the total area growth strategy, the unstratified cohort had a mean bias of 0.08 (95% CI: –0.05 to 0.22) and limits of agreement ranging from –1.15 (95% CI: –1.36 to –0.93) to 1.32 (95% CI: 1.01 to 1.61) (Fig 3A). Similar values were seen for the small lesion cohort (<0.9 DA) with an intereye bias of 0.05 (95% CI: –0.10 to 0.20) and limits of agreement ranging from –0.79 (95% CI: –1.02 to –0.58) to 0.89 (95% CI: 0.59 to 1.13) (Fig 5A). Eyes with baseline hyperTD area ≥0.9 DA demonstrated a bias of –0.01 (95% CI: –0.18 to 0.17), with corresponding lower and upper limits of agreement of –1.30 (95% CI: –1.60 to –0.98) and 1.28 (95% CI: 0.93 to 1.66), respectively (Fig 5D).

Using the sqrt area growth strategy, we found the bias remained low with negligible differences between the unstratified, small, and large lesion groups. The unstratified group had an intereye bias of 0.015 (95% CI: –0.02 to 0.05) and limits of agreement from –0.33 (95% CI: –0.39 to –0.26) to 0.36 (95% CI: 0.28 to 0.45) (Fig 3B). Eyes with baseline hyperTD area <0.9 DA (Fig 5B), demonstrated an intereye bias of 0.0008 (95% CI: –0.06 to 0.06), with limits of agreement ranging from –0.37 (95% CI: –0.47 to –0.27) to 0.38 (95% CI: 0.29 to 0.46). In eyes with baseline hyperTD area ≥0.9 DA (Fig 5E), the bias was –0.01 (95% CI: –0.04 to 0.02), with corresponding lower and upper limits of agreement of –0.23 (95% CI: –0.29 to –0.17) and 0.22 (95% CI: 0.15 to 0.30), respectively. The larger lesion subgroup showed an improvement with narrower limits of agreement.

**Figure 4:**
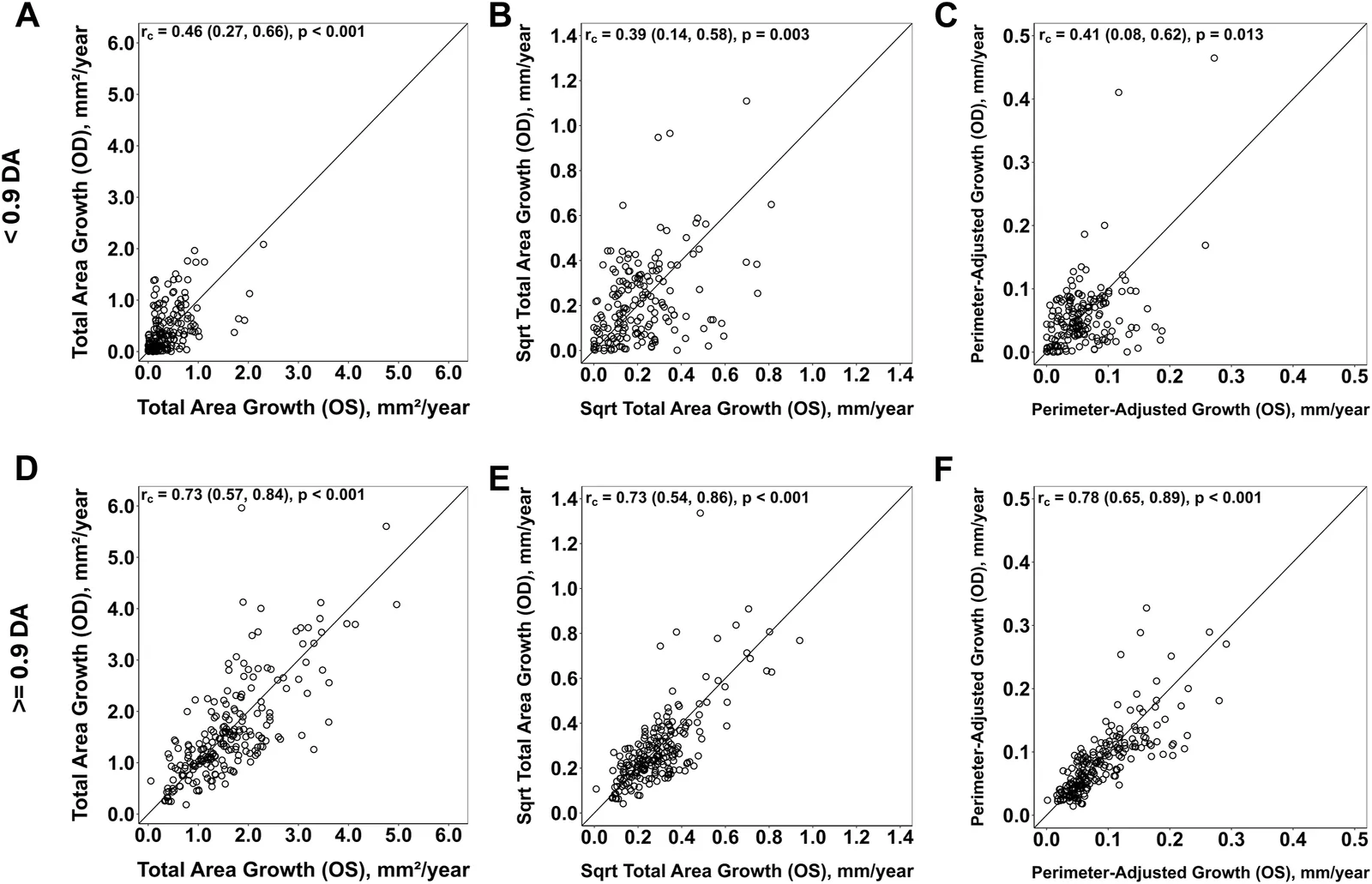
Scatterplots showing intereye concordance in annualized growth rates of large hyperTDs stratified by baseline lesion area. The y-axis represents growth rates for the OD, and the x-axis represents those for the OS. The diagonal line denotes the line of equality (slope = 1), indicating perfect agreement between OD and OS and a concordance correlation coefficient (r_c_) of 1. Each point represents an annualized growth-rate interval of 1 year ± 1 month. Confidence intervals and *P* values for Lin concordance correlation coefficients were obtained using cluster bootstrap resampling. **A-C,** Intereye concordance for annualized growth-rate intervals with bilateral baseline lesion size <0.9 DAs. **D-F,** Those with baseline lesion size ≥0.9 DAs. **A, D**, Scatterplots for the total area growth strategy; **B, E,** scatterplots for the square root (sqrt) area growth strategy; and **C, F,** scatterplots for the perimeter-adjusted growth strategy. The data points are more closely aligned with the line of unity for baseline size ≥0.9 DA (**D-F**) compared to **A-C**. Quantitatively, the concordance coefficient shows a significant but weak concordance for smaller lesions (<0.9 DA), (**A**, r_c_ = 0.46, *P* < 0.001; **B**, r_c_ = 0.39, *P* = 0.003; **C**, r_c_ = 0.41, *P* = 0.013), whereas a statistically significant and strong concordance is observed for larger lesions (≥0.9 DA) with comparable coefficients across all 3 growth strategies (**D**, r_c_ = 0.73; **E**, 0.73; **F**, 0.78, all *P*s < 0.001). DA = disc area; hyperTD = hypertransmission defect; OD = right eye; OS = left eye.

**Figure 5:**
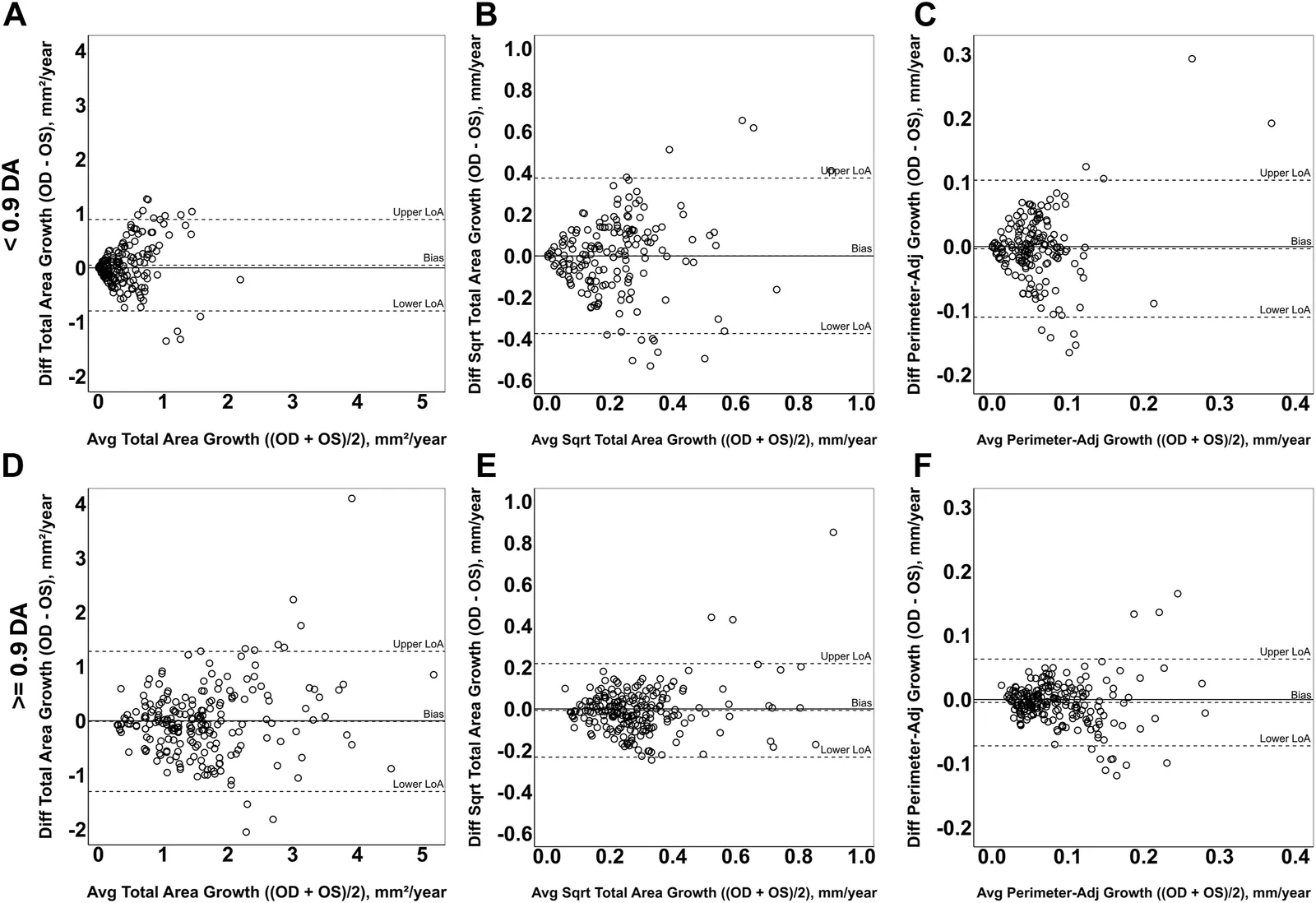
Bland–Altman plots showing intereye agreement in annualized GA growth rates stratified by baseline lesion area. The y-axis shows the difference in growth rate between the OD and OS, the x-axis represents the mean growth rate of both eyes. Each point represents an annualized growth-rate interval of 1 year ± 1 month. The central dashed line (“Bias”) denotes the mean intereye difference. The upper and lower dotted lines (“Upper LoA” and “Lower LoA”) represent the 95% limits of agreement, calculated as Bias ± 1.96 × SD_differences_. **A, D,** Results for the total area growth strategy; **B, E,** results for the square root (sqrt) area growth strategy; and **C, F,** results for the perimeter-adjusted area growth strategy. **A-C**, All growth intervals with baseline hyperTD area <0.9 DAs (equivalent to 2.29 mm^2^), whereas **D-F** include intervals with baseline lesion area ≥0.9 DAs. Because the same units are used, direct comparison of the OD–OS agreement across baseline size categories is possible within each growth-rate strategy (**A vs. D**, **B vs. E**, **C vs. F**). When comparing the LoA for different growth-rate strategies for both the smaller and larger lesions, we found the LoA to be narrower for the sqrt and perimeter-adjusted growth-rate strategies for the larger lesions; in contrast, the LoA were narrower for the smaller lesions when using the area growth-rate strategy, which did not correspond to greater concordance/tighter clustering around the line of unity in Figure 4 A as it did with 4 E, F. DA = disc area; GA = geographic atrophy; hyperTD = hypertransmission defect; LoA = limits of agreement; OD = right eye; OS = left eye; SD = standard deviation.

A comparable pattern was observed with the perimeter-adjusted growth strategy. The unstratified cohort showed a bias of –0.0003 (95% CI: –0.01 to 0.01) and limits of agreement from –0.10 (95% CI: –0.11 to –0.07) to 0.09 (95% CI: 0.07 to 0.12) (Fig 3C). Eyes with baseline hyperTD area <0.9 DA (Fig 5C) showed a bias of –0.003 (95% CI: –0.02 to 0.01), with limits of agreement from –0.11 (95% CI: –0.14 to –0.08) to 0.10 (95% CI: 0.08 to 0.14). In eyes with baseline hyperTD area ≥0.9 DA (Fig 5F), the bias was –0.005 (95% CI: –0.02 to 0.01), with lower and upper limits of agreement of –0.07 (95% CI: –0.10 to –0.05) and 0.06 (95% CI: 0.04 to 0.08), respectively. The bias was minimal and negligibly different between the subgroups, but the limits of agreement were narrower for the larger lesions. Notably, all the CIs for the bias values included zero, indicating no significant systematic bias in any of the 3 growth outcomes for the unstratified and stratified cohorts. However, as shown in the Bland–Altman plots from Figures 3 and 5, there is evidence of proportional bias (i.e., increasing differences in hyperTD growth between OD and OS with increasing average hyperTD growth of OD and OS) for all baseline hyperTD areas. The totality of the symmetry analyses indicates that the intereye symmetry, as assessed by concordance correlation coefficients and Bland–Altman plots, is better for the larger baseline lesions regardless of the growth-rate measurement strategy. This is visually evident by appreciating the tighter clustering of the data points around the lines of equality with essentially zero bias observed for the sqrt and the perimeter-adjusted growth-rate strategies in Figures 4E, F and 5E, F respectively.

When we calculated the required sample sizes for a 2-arm, 1-year RCT for each growth-rate outcome for various lesion sizes, there was a noticeable advantage with respect to the required sample sizes for sqrt total area growth rate and perimeter-adjusted growth rate compared with the total area growth rate for bilateral initial lesions sizes <0.9 DA, but that advantage largely disappeared for bilateral initial lesion sizes ≥0.9 DA and the entire cohort of all bilateral initial lesion sizes. Moreover, using a paired-eye trial design with fellow eyes randomized to different arms greatly reduces the total number of patients needed across initial lesion sizes. For initial lesions sizes 1-7 DA, which is the standard inclusion criterion for past clinical trials, only 15, 13, and 16 total patients would be required for the total area growth, sqrt area growth, and perimeter-adjusted growth outcomes, respectively, versus 122, 108, and 120 total patients for an unpaired trial with only 1 eye enrolled per patient (Table 2). For a systemic therapy that would affect both eyes in which both eyes are included in either the active treatment or control arms, we found that 95, 88, and 123 total patients would be required for the total area growth, sqrt area growth, and perimeter-adjusted growth outcomes, respectively, versus 122, 108, and 120 total patients for an unpaired trial with only 1 eye enrolled per patient (Table 3).

**Table 2:** Sample Size Calculations for a 2-Arm, Equal Allocation, 1-Year Treatment Trial Using Both Eyes of a Patient Randomized to Different Treatment Arms or 1 Eye of a Patient Randomized to a Treatment Arm to Show Reduction in the Growth Rates of Large hyperTDs

Subset of Annual ER Measurements	Total Area Growth Rate (mm^2^/yr)		Sqrt Total Area Growth Rate (mm/yr)		Perimeter-Adjusted Growth Rate (mm/yr)	
	Total Patients Needed across Arms for a Paired 2-Eye Trial∗‡	Total Patients Needed across Arms for an Unpaired 1-Eye Trial†‡	Total Patients Needed across Arms for a Paired 2-Eye Trial∗‡	Total Patients Needed across Arms for an Unpaired 1-Eye Trial†‡	Total Patients Needed across Arms for a Paired 2-Eye Trial∗‡	Total Patients Needed across Arms for an Unpaired 1-Eye Trial†‡
All§	32	350	40	196	38	198
Baseline lesion size <0.90 DA‖	92	514	71	298	79	286
Baseline lesion size ≥0.90 DA¶	18	136	17	112	17	122
Baseline lesion size 1-7 DAs#	15	122	13	108	16	120

DA = disc area; ER = enlargement rate; hyperTD = hypertransmission defect; Sqrt = square root.

∗ Assuming a normal distribution for the difference between the treated and untreated eyes, 2 eyes enrolled per participant, a paired *t* test, 80% power to detect a 30% reduction in ERs in the treatment arm compared to the untreated arm, 2-sided α = 0.05 (i.e., type I error = 5%), and 1:1 treatment allocation with fellow eyes randomized to different arms.

† Assuming a normal distribution for the difference between treated and untreated eyes, 1 eye enrolled per participant, a 2-sample *t* test, 80% power to detect a 30% reduction in ERs in the treatment arm compared to the untreated arm, 2-sided α = 0.05, and 1:1 treatment allocation.

‡ These numbers are not adjusted for dropout. To adjust for a dropout proportion of d, divide these numbers by (1 – d) and round up (i.e., n_d_ = ceiling(n/(1 – d))).

§ Based on 942 annual ER measurements from 128 eyes of 64 subjects for the paired trial and 1868 annual ER measurements from 221 eyes of 157 subjects for the unpaired trial.

‖ Based on 348 annual ER measurements of 66 eyes of 33 subjects for the paired trial and 1006 annual ER measurements of 147 eyes of 111 subjects for the unpaired trial.

¶ Based on 438 annual ER measurements of 68 eyes of 34 subjects for the paired trial and 862 annual ER measurements of 117 eyes of 82 subjects for the unpaired trial.

# Based on 400 annual ER measurements from 68 eyes of 34 subjects for the paired trial and 795 annual ER measurements from 113 eyes of 78 subjects for the unpaired trial.

**Table 3:** Sample Size Calculations for a 2-Arm, Equal Allocation, 1-Year Treatment Trial Using Both Eyes of a Patient Randomized to the Same Treatment Arm or 1 Eye of a Patient Randomized to a Treatment Arm to Show Reduction in the Growth Rates of Large hyperTDs

Subset of Annual ER Measurements	Total Area Growth Rate (mm^2^/yr)		Sqrt Total Area Growth Rate (mm/yr)		Perimeter-Adjusted Growth Rate (mm/yr)	
	Total Patients Needed across Arms for a 2-Eye Trial∗‡	Total Patients Needed across Arms for a 1-Eye Trial†‡	Total Patients Needed across Arms for a 2-Eye Trial∗‡	Total Patients Needed across Arms for a 1-Eye Trial†‡	Total Patients Needed across Arms for a 2-Eye Trial∗‡	Total Patients Needed across Arms for a 1-Eye Trial†‡
All§	218	350	118	196	142	198
Baseline lesion size <0.90 DA‖	243	514	157	298	183	286
Baseline lesion size ≥0.90 DA¶	100	136	94	112	121	122
Baseline lesion size 1-7 DAs#	95	122	88	108	123	120

DA = disc area; ER = enlargement rate; hyperTD = hypertransmission defect; Sqrt = square root.

∗ Assuming a normal distribution for the difference between treated and untreated eyes, both eyes of each participant randomized to the same treatment (i.e., randomization occurs at the patient and not the eye level), a 2-sample *t* test, 80% power to detect a 30% reduction in ERs in the treatment group compared with the untreated group, 2-sided α = 0.05, and 1:1 treatment allocation.

† Assuming a normal distribution for the difference between treated and untreated eyes, 1 eye enrolled per participant, a 2-sample *t* test, 80% power to detect a 30% reduction in ERs in the treatment arm compared to the untreated arm, 2-sided α = 0.05, and 1:1 treatment allocation.

‡ These numbers are not adjusted for dropout. To adjust for a dropout proportion of d, divide these numbers by (1 – d) and round up (i.e., n_d_ = ceiling(n/(1 – d))).

§ Based on 942 annual ER measurements from 128 eyes of 64 subjects for the 2-eye trial and 1868 annual ER measurements from 221 eyes of 157 subjects for the 1-eye trial.

‖ Based on 348 annual ER measurements of 66 eyes of 33 subjects for the 2-eye trial and 1006 annual ER measurements of 147 eyes of 111 subjects for the 1-eye trial.

¶ Based on 438 annual ER measurements of 68 eyes of 34 subjects for the 2-eye trial and 862 annual ER measurements of 117 eyes of 82 subjects for the 1-eye trial.

# Based on 400 annual ER measurements from 68 eyes of 34 subjects for the 2-eye trial and 795 annual ER measurements from 113 eyes of 78 subjects for the 1-eye trial.

## Discussion

In this study, we investigated intereye symmetry of GA growth in patients with bilateral hyperTDs spanning a wide range of baseline lesion sizes (0.04 mm^2^ to 30.30 mm^2^). We built on the methodology described by Herrera et al^12^ and compared annualized growth rates between eyes using 3 established growth-rate strategies.^6^^,^^11^ When intervals across all baseline lesion sizes were compared between OD and OS, we observed a high intereye concordance using the total area growth strategy (r = 0.76). However, lower concordance was observed with the sqrt and perimeter-adjusted strategies (r = 0.51 and 0.60, respectively). In a secondary analysis, we sought to understand whether these lower concordances were a result of an inappropriately selected growth-rate strategy. As Herrera et al^12^ indicated, either the sqrt area growth-rate strategy or the perimeter-adjusted growth-rate strategy is best suited to lesions <0.9 DA, whereas any of the total area, sqrt area, or perimeter-adjusted growth rate strategies can be used for lesions above this cutoff. We therefore reapplied the 3 growth-rate strategies in subcohorts stratified according to the baseline hyperTD area breakpoint of 0.9 DA. Contrary to our expectations, this stratification revealed that the overall reduction in concordance was not an effect of the growth-rate transformation strategy but largely driven by smaller lesions (<0.9 DA), which exhibited weaker concordance across all 3 growth-rate strategies (r_c_ = 0.39 to 0.46). In contrast, larger lesions (≥0.9 DA) demonstrated consistently strong intereye concordance across all growth-rate strategies (r = 0.73 to 0.78).

Bland–Altman analyses of intereye agreement showed narrower limits of agreement and smaller standard deviations of the difference (OD - OS) for the total area growth-rate strategy for the smaller lesion cohort, but this did not correspond to greater concordance between OD and OS for total area growth rate. In contrast, the sqrt growth and perimeter-adjusted growth-rate strategies demonstrated narrower limits of agreement and lower standard deviation in the larger lesion cohort, with tighter clustering of data points around the line of equality between OD and OS, indicating improved agreement with the larger lesions. Taken together, these findings confirm the presence of good intereye symmetry in growth of hyperTDs only after both eyes have reached a hyperTD area of ≥0.9 DA.

One of the earliest landmark studies on GA symmetry by Sunness et al^3^ reported an intereye correlation coefficient of 0.76, matching our initial result when using total area growth in the unstratified cohort. Similarly, Fleckenstein et al^38^ reported a concordance coefficient of 0.756, representing another almost identical finding. Higher concordance values have been reported by Klein et al^39^ (0.85) in 12 bilateral cases followed over a 5-year duration, by Colijn et al^5^ (0.87), and the highest value from our literature search by Lindblad et al^40^ with 0.88 in a cohort of 118 patients. In light of these consistently high estimates, reviews and meta-analyses have concluded that GA progression exhibits strong natural intereye symmetry, thereby supporting the use of fellow eye as a control in clinical trials, with the potential benefits of reduced sample size, cost, and study duration.^10^^,^^41^

However, the aforementioned studies primarily based their conclusions on measurements of total area growth and using lesions >0.9 DAs. Moreover, in a reanalysis of the Age-Related Eye Disease Studies data, Feuer et al^11^ reported that the total area growth strategy was dependent on baseline lesion size, an effect which could be reduced by applying a sqrt transformation. Subsequently, Shen et al^6^ showed that GA growth is also dependent on baseline lesion perimeter, focality, and circularity, factors not accounted for by the sqrt approach, and they proposed the perimeter-adjusted growth rate to address these limitations.

Herrera et al^12^ subsequently evaluated the appropriateness of each growth-rate strategy in a cohort of over 200 eyes with hyperTDs using 1867 annualized growth rates with baseline lesion sizes ranging from 0.04 mm^2^ to 51.62 mm^2^ as measured from *en face* SS-OCT images. For the longitudinal follow-up of these hyperTDs, Herrera et al^12^ found that there was a breakpoint in lesion sizes where different growth-rate strategies should be used to eliminate the impact of baseline lesion size on the growth rates. They found that a breakpoint of 0.9 DA should be used to stratify lesions, and either the sqrt area growth rate or the perimeter-adjusted growth rate was best for following these smaller lesions. For lesions measuring ≥0.9 DA, any of the total area, sqrt area, or perimeter-adjusted growth rates can be used for following these hyperTDs that tended to be more circular and unifocal.

The symmetry reported in prior studies appeared to be largely based on cohorts overrepresented by large lesions. Sunness et al^3^ reported that only 6% of eyes had baseline hyperTD area <0.5 DA, and based on their tabular and graphical data, we can infer about 15% of the lesions were below the 0.9 DA threshold. Similarly, descriptive statistics reported by Fleckenstein et al^38^ suggest that <15% of eyes would fall in the small lesion cohort. Additionally, these studies relied on measurements of GA derived from CFI and FAF imaging. While CFI has been historically used for diagnosing and longitudinal monitoring of AMD and GA, CFI lacks sufficient contrast to reliably detect and delineate early or small areas of atrophy.^17^ As a result, many natural history studies, as discussed earlier, likely underrepresented early and small lesions. Of note, once we restricted our analyses to the larger lesions that were studied previously, we found high concordance between eyes. Moreover, the concordance was similar with each of the growth-rate strategies, but the variability or the limits of agreement between the 2 eyes were narrower when using the sqrt or perimeter-adjusted growth-rate strategies, which suggests that both of these strategies control for baseline lesions characteristics such as lesion size that can influence the growth rate of GA or large hyperTDs.

Additionally, we used our findings to calculate sample sizes for powering a clinical trial to detect a 30% reduction in hyperTD growth rate. While the smaller lesion cohort required the largest sample size associated with lower concordance and higher variability in baseline features such as circularity and focality, we found that application of the sqrt area growth and perimeter-adjusted growth showed the greatest advantage in this cohort. Nevertheless, regardless of the growth strategy or baseline size, in a paired-eye trial design in which 1 eye is treated and the fellow eye served as control, or in a 2-eye trial using systemic therapy in which both eyes serve as treated eyes or control eyes, there were markedly reduced recruitment requirements when compared with an unpaired study design when only 1 eye is enrolled per patient with the notable exception of perimeter-adjusted growth for larger lesions in a 2-eye trial using systemic therapy. Even with zero intereye correlation, a paired-eye design would only require half as many patients as a conventional unpaired 2-arm trial, which may not always be feasible. However, the primary advantage of identifying subgroups with higher symmetry and concordance lies in the substantial reduction in the variance of the within-patient differences. This markedly improves statistical power, as demonstrated in one scenario from Table 2 where the required sample size decreased from 108 patients in an unpaired design to as few as 13 patients.

This benefit of using fellow eyes, especially in the paired-eye control group design, likely stems from the ability to isolate treatment effects by controlling for shared genetic and nongenetic systemic environmental influences. Additionally, our analyses of growth-rate concordance among unrelated paired eyes indicated that the strong concordance observed between fellow eyes exceeded what would be expected from baseline lesion characteristics alone, supporting the role of shared genetic and environmental factors in GA progression. However, the observed lower concordance among lesions of smaller baseline sizes may indicate the persistence of nongenetic confounders that influence disease progression such as the variability of hyperTD onset and perfusion differences between eyes that would impact the lesion’s multifocality, perimeter, and noncircularity.^42^^,^^43^

An important point of consideration in clinical trial recruitment is identification of fast versus slow progressors. In our Bland–Altman analysis, there was evidence of proportional bias for all 3 growth outcomes, albeit to a lesser degree for baseline lesions ≥0.9 DA or 1-7 DA. Because the absolute intereye differences in growth rate increased with increasing average growth rate, cohorts enriched with fast progressors may be associated with greater variability. However, we observed that the ratios of the means to standard deviations of the differences (OD – OS) for fast progressors tended to be greater than the ratios in the entire cohorts, which would reduce the required sample sizes. In contrast, slower-progressing eyes tended to have ratios that were lower, which would increase the required sample size. This heteroscedasticity will not be an issue in future trials provided randomization balances the distributions of fast and slow-growing lesions in each treatment arm, which is what we anticipate from current clinical trial randomization. Moreover, if studies only recruit fast progressors then our current recruitment estimates should suffice; the proportional bias for all 3 growth outcomes should not increase the required sample sizes.

A key strength of our study is the ability to detect and longitudinally track the earliest onset of atrophy known as large hyperTDs.^12^ With OCT imaging now the gold standard for imaging AMD^13^^,^^14^^,^^22^ and the growing trend to treat irreversible macular atrophy as early as possible, it has become important to detect the appearance and growth of these large hyperTDs over a wide range of lesion sizes.^12^^,^^44^, ^45^, ^46^ Our *en face* imaging strategy based on high-density raster scans allows for the detection of these atrophic regions by using choroidal hyperTDs detected on the sub-RPE slab in conjunction with corresponding B-scans.^12^^,^^22^^,^^23^ By improving the detection of large hyperTDs, our data set is better suited to capture the variability and complexity of size, border configuration, perimeter, multifocality, and circularity among small lesions. As a result, we were able to reveal markedly reduced intereye symmetry with smaller lesions that has gone undetected in prior characterizations of natural GA progression. Moreover, another strength of our study is an annotation protocol that ensures reliability of results using a 2-grader assessment of all scans and the use of a validated semiautomated algorithm, with manual review for each scan and correction performed as needed. Another strength of our analysis lies in implementation of an established size-based stratification previously investigated by Herrera et al.^12^ We used a subcohort consisting exclusively of bilateral GA cases from the larger natural history cohort described by Herrera et al,^12^ where the authors derived the 0.9 DA cutoff using piecewise linear mixed modeling to categorize GA growth-rate trends. The current study applies the 0.9 DA cutoff in a distinct context that evaluates intereye concordance in growth rates rather than comparing the growth-rate behavior itself, which further validates the utility of this cutoff threshold.

A recent publication evaluating intereye correlations for GA growth rates in fellow eyes revealed only a modest correlation in their patient cohort.^47^ Contrary to our results, these findings were observed across all 3 growth-rate metrics and GA size categories. We believe that the differences in results exemplify the differences in the patient populations and methodology studied in these reports. Firstly, our hyperTD annotation strategy with en face and B-scan SS-OCT imaging would be considered the gold standard for assessing lesion size and configuration, and more accurate than CFI,^15^, ^16^, ^17^ and our data set contained a wide range of lesion sizes, especially with a greater representation of smaller lesions as 348 of the 786 paired intervals belonged to the size category <0.9 DA, compared with the curated Age-Related Eye Disease Study 2 data set in which very small lesions would be particularly difficult to assess. In addition, the Age-Related Eye Disease Study 2 database was not representative of patients undergoing routine clinical care, but rather, a curated collection of patients that were enrolled with large drusen or nonfoveal GA in both eyes or large drusen or nonfoveal GA in 1 eye and advanced AMD in the fellow eye defined by foveal involvement. Consequently, compared with our database of eyes in this report, the eyes studied by von der Emde et al^47^ do not represent a population found in routine clinical practice and should not have been used for symmetry studies. Similarly, the cutoffs used to define the small (<0.75 DA), medium (0.75-1.5 DA), and large (>1.5 DA) subcohorts are not practically useful for enrollment in clinical trials compared with our threshold of 0.9 DA, which is very similar to the current clinical trial inclusion criteria of 1-7 DA.

Limitations of our methodology include the “rolling” annual growth rate from lesions followed over many years as the size of the large hyperTDs increased, which necessarily involved only considering pairs of observations from the same eye separated by 1 year ± 1 month that had an exactly matching interval in the fellow eye. In an RCT with defined study visits, this would not be necessary. In any case, cluster bootstrap resampling with patients as the resampling unit was used to account for clustering of fellow eyes as well as repeated measurements from the same eye to obtain valid statistical inference.^12^ Additionally, the SS-OCT imaging device used in this study is not widely available in a clinical setting; however, prior studies using dense raster spectral-domain OCT imaging have shown reproducible results for hyperTD annotation.^48^

Lastly, we cannot entirely exclude measurement error as a source of variability. Yehoshua et al^49^ showed, using the absolute area growth-rate strategy, both the test–retest and inter-reader absolute variability for annotation of atrophy on *en face* OCT images increased with lesion size, but the slope of this linear relationship is <1; therefore, we can conclude from their graphical representation that the relative variability is greater in smaller lesions. However, this baseline area dependence disappeared after using the sqrt transformation of the lesion area. Similarly, the perimeter-adjusted strategy, which accounts for greater perimeter length in larger lesions, further minimizes boundary-related sources of error. Application of growth-rate transformations will remove the dependence of absolute variability on lesion area, subsequently stabilizing relative variability across small and large lesions. Although a restricted range of growth rates can attenuate concordance coefficients, lesions <0.9 DA in our cohort exhibited relatively high variability in growth rates. Previous studies have shown that lesions <2.54 mm^2^ (1 DA) display greater variability in growth rates than lesions ≥2.54 mm^2^.^12^ Consequently, the lower concordance observed in smaller lesions (<0.9 DA) is most likely attributable to the biology of growth rather than methodological artifacts.

In summary, this study investigated the intereye symmetry of large hyperTD growth using 3 established growth-rate strategies. When lesions across all baseline sizes were analyzed together, we observed high intereye correlation using the total area growth-rate strategy but weaker concordances with the sqrt area and perimeter-adjusted strategies. However, when we stratified by baseline lesion sizes using our previously published breakpoint of 0.9 DA, the intereye concordance correlation coefficients of growth rates were similar when using any of the growth-rate strategies, with the best concordance observed for the larger lesions and the least measurement variability observed when using the sqrt and perimeter-adjusted growth-rate strategies. By applying 3 growth-rate strategies, previously untested in bilateral analyses, in conjunction with a baseline size breakpoint, we revealed a difference in concordance between small and large lesions that had not been previously recognized. These observations will help inform the inclusion criteria for trials investigating therapies for GA or hyperTDs in which 1 eye is treated and the fellow eye serves as a control. These findings especially support the use of fellow eyes as controls in clinical trials when both eyes have a baseline hyperTD area of ≥0.9 DA.
